# The Gut Microbiome and Its Implication in the Mucosal Digestive Disorders

**DOI:** 10.3390/biomedicines10123117

**Published:** 2022-12-02

**Authors:** Laura Bozomitu, Ingrith Miron, Anca Adam Raileanu, Ancuta Lupu, Gabriela Paduraru, Florin Mihai Marcu, Ana Maria Laura Buga, Daniela Carmen Rusu, Felicia Dragan, Vasile Valeriu Lupu

**Affiliations:** 1 Pediatrics Department, “Grigore T. Popa” University of Medicine and Pharmacy, 700115 Iasi, Romania; 2 Faculty of Medicine and Pharmacy, University of Oradea, 410087 Oradea, Romania; 3 Faculty of Medicine, “Grigore T. Popa” University of Medicine and Pharmacy, 700115 Iasi, Romania

**Keywords:** microbiome, human, digestive pathology, dysbiosis, probiotics

## Abstract

The gastrointestinal (GI) tract is one of the most studied compartments of the human body as it hosts the largest microbial community including trillions of germs. The relationship between the human and its associated flora is complex, as the microbiome plays an important role in nutrition, metabolism and immune function. With a dynamic composition, influenced by many intrinsic and extrinsic factors, there is an equilibrium maintained in the composition of GI microbiota, translated as “eubiosis”. Any disruption of the microbiota leads to the development of different local and systemic diseases. This article reviews the human GI microbiome’s composition and function in healthy individuals as well as its involvement in the pathogenesis of different digestive disorders. It also highlights the possibility to consider flora manipulation a therapeutic option when treating GI diseases.

## 1. Introduction

It is known that humans are living ecosystems including human cells as well as microorganisms. The population of microorganisms found in our body or on its surface consists of bacteria, viruses, fungi and protozoa, and it is called microbiota, while the term “microbiome” is used to describe their genomes [1,2].

The human microbiome can be found on the skin, in the oral cavity, in the gastrointestinal (GI) tract, the respiratory tract, and the genitourinary tract, including epithelial barriers and body fluids; however due to the bioavailability of nutrients; the largest concentration and diversity of microbiota can be found in the GI tract. Studies estimate that the human GI microbiota accounts for about 10^12^ microbes per gram of content, representing around 5000 distinct species [2,3]. Interactions between the human and its associated microbiota involve numerous complexities with implications in various immunological, neuronal, metabolic and endocrine responses attained through multiple biological systems such as the intestinal neuro-immune axis [2,3,4,5].

The medical literature illustrates the idea that all individuals share a core microbiome; however, each individual has a unique composition and diversity of microbes [6]. Although the microbiome is dynamic, changing in relation with human age and health status, there is an equilibrium between different types of species maintaining eubiosis and sustaining an absence of pathology [1,3]. “All diseases begin in the gut” is an ancient quote that still maintains its truth: alteration of the composition and function of the healthy microbial structure leads to dysbiosis, resulting in various GI disorders, systemic metabolic diseases, and neurological impairments. Many of these affections depend on their metabolic programing early in life and can be alleviated or prevented through early intervention via flora disruption [7,8].

Besides bacteria, the human body is host to an impressive amount of viruses, their number exceeding even 10 times the number of commensal bacteria [9]. The human virome has a specific site composition over different anatomical segments of the human body. The largest number of viruses is found in the GI tract. The GI virome has a highly individualized composition, depending on age, diet, lifestyle and geographical location. Its composition consists of both DNA and RNA viruses, in addition to prokaryotic and eukaryotic viruses. Similar to human microbiota, the human GI virome composition and diversity changes over time, evolving gradually to its stable adult form. Infants within their first days of life are characterized by high phageome and a low bacteriome diversity, shifting to a low phageome and a high bacteriome diversity across the age of two years [9,10,11].

In the past, fungi were studied as individual species as they were considered human pathogens. Current studies showed that fungi are a component of the human body’s ecosystem, described as the human “mycobiome”. Similar to the bacterial and viral microbiome, the mycobiome has a variable composition shaped by several factors [12]. The presence of the mycobiome has been detected in the gut of at least 70% of healthy adults [13]. *Ascomycota* is the most prevalent phylum, covering 48% to 99% of all present species. In contrast, *Basidiomycota* is the less abundant phylum, ranging between 0.5% to 14% of identified microscopic fungi, followed by the phylum *Mucoromycota* [14,15]. Two mycotypes can be distinguished in the gut. Mycotype 1 is defined by a high abundance of the genus Saccharomyces and also other unclassified genera, while mycotype 2 consists of the *genera Penicillium*, *Malassezia* and *Mucor* [12].

Recent advancements in molecular techniques proved that the human microbiome may play an important role in health and disease progression. In this article, we attempt to correlate the complex relationships between the microbiome and the digestive pathology from the disease inception to therapeutic intervention using the immune modulation of the microbiota.

## 2. Microbiota in Healthy Individuals

The gastrointestinal tract harbors the largest number of microorganisms from the human body, as it offers a favorable habitat and plentiful nutrients for a great diversity of microbial species. Concurrently, the GI represents the site of the most extensive network of communication systems between the commensal flora and the immune system. The acquired and the innate immunity ensure the immune homeostasis and tolerance for the GI flora [5,16,17].

GI flora is represented by five primary bacteria phyla: Firmicutes (synonym Bacilliota) and Bacteroides (synonym Bacteroidota) phylum predominate the microbiome, while Actinobacteria (synonym Actinomycetota), Proteobacteria (synonym Pseudomonadota) and Verrucomicrobia phylum are found in modest proportions, similar in healthy adults, however there are notable changes in the interindividual variation of genus and species, conditioned by genetic and environmental factors [1,18].

Unlike the human genome, which is inherited from siblings, the human microbiome is something we acquired and changes its composition during one’s existence. For decades it was considered that the uterus is sterile; however, recent studies have proven that the development of the microbiome starts during prenatal life and continues during birth, breastfeeding and throughout senescence [2,3,19,20]. Pre- and postnatal microbial stimulation is essential for developing T-helper type 1 (Th1) and regulatory-T cells (Treg) mediated immune responses [21,22,23] The infant’s future microbiome depends on his mother’s gut and urogenital flora, however the mechanisms that ensure the passing of the microbes from mother to fetus are not fully understood. Studies analyzing infants’ meconium, the first stool after birth, were found to have no difference in bacterial composition, regardless of their delivery method. On the contrary, when compared to placental and amniotic fluid, there was an approximately 50% percent matching with meconium microbes, probably as a result of amniotic fluid ingestion during pregnancy and in utero colonization [19].

Babies born naturally through the birth canal have a microbiota similar to their mother’s vaginal and fecal microbiota (*Lactobacillus* and *Bifidobacterium* genera), while infants delivered through cesarean section receive germs from their mother’s skin and the surrounding environment (*Staphylococcus*, *Corynebacterium*, and *Propionibacterium* genera). Regarding the time of delivery, preterm infants’ microbiota is characterized by a reduction in *Bifidobacterium* and *Bacteroides* species with a secondary increase in the number of potentially pathogenic bacteria [2].

During pregnancy, different factors such as the mother’s diet, antibiotic exposure, stress and health status seem to have a certain influence on the fetuses early colonization and state of well-being [2]. Maternal obesity as well as an inappropriate diet leads to a reduction in infant gut bacteria, with low levels of *Bifidobacterium*, similar to the microbiota of obese adults [19].

There is an increased risk of overweight, asthma and inflammatory bowel disease (IBD) in childhood linked to an early exposure to antibiotics prenatally and in infancy [2,19,22]. The early administration of antibiotics leads to lower levels of *Bifidobacterium* and *Lactobacillus* genera, while pathogens such as *Staphylococcus*, *Streptococcus*, *Serratia*, and *Parabacteroides* genera increase in number. A decrease of 25% in microbiota diversity has been identified after only 7 days of antibiotic use. Based on their spectrum of antimicrobial intensity, some antibiotics are associated with more than 2 years of disruptive damages in the gut microbial environment: clindamycin decreases *Bacteroides* diversity, clarithromycin and ciprofloxacin have a similar effect on *Actinobacteria* and *Ruminococcus* spp., while vancomycin reduces *Bacteroides*, *Ruminococcus* and *Faecalibacteria* populations [24]. Antibiotics resistance genes, found in the maternal microbiome, were also identified in infant perinatal stool samples. A stress related maternal microbiome, it is associated with higher rates of allergy and gastrointestinal issues in infants and lower rates of *Bifidobacterium* and *Lactobacillus* spp. in their gut [19].

Apart from the mother’s influence during pregnancy and early childhood, genetics seem to be responsible for similarities found between family members’ microbiota. As an example, when compared to dizygotic twins, monozygotic twins tend to have more similar features regarding their GI microbiota. Children with siblings are more likely to have more *Bifidobacterium* than families with a single child [2,4,18].

Feeding practices also play a major role in the development of the infant’s microbiota. *Enterococcus*, *Enterobacteriaceae*, *Bacteroides*, *Clostridium* and *Streptococcus* genera are dominating the flora of formula-fed infants. Breast milk, through its nutrients and bioactive compounds, promotes the growth of beneficial bacteria like lactobacilli and bifidobacteria, encouraging healthy immune function as an important supporter of the child’s health condition. When shifting from milk to solid foods, a toddler’s microbiota becomes enriched, with *Bacteroidota* and *Bacilliota phylum* dominating the gut [2,24]. In children between 2–5 years of age, it is observed that a stable adult-like microbiota is achieved, although the microbial composition continues to gradually enrich its diversity until the age of 7–12 years, with *Bacilliota* and *Actinomycetota* phylum in greater proportion than that found in adult microbiota. As children age, their microbiota diversifies, gradually achieving a the status similar to adult microbiota [4].

Geographical distribution, with its locally inclined food consumption cultures and preferences, dietary habits such as high levels of saturated fat, sugar and a low fiber intake have been linked to a pro-inflammatory microbiota with reduced diversities. Alcohol and tobacco use and a sedentary lifestyle negatively influence the GI microbiota. Socioeconomic status, pollution and household pets also interfere with normal microbiome composition [4,18,25].

## 3. Microbiota and the Digestive Pathology

We will describe the major microbial shifts related to different GI diseases and the therapeutic option available for restoring the normal flora using probiotics. Probiotics are defined by World Health Organization (WHO) as living microorganisms that have positive effects on the host when administrated in the right amounts [26]. Recently they received immunomodulatory proprieties as they influence GI homeostasis and modulate the systemic and mucosal immunity [23,27,28]. As for their beneficial outcomes, we can also mention the ability of regulating the disrupted intestinal flora, the protection of the epithelial barrier integrity, the ability to inhibit the adhesion of pathogenic flora through competition, their encouragement of the production of mucin, B-cell-secreting IgA, as well as short chain fatty acids (SCFAs) with immune modeling and anti-inflammatory effects [29].

### 3.1. Esophageal Pathology

The esophageal microbiome in healthy individuals is complex but with relatively constant composition across the upper, middle and lower segments of the esophagus [30]. It includes six phyla with the predominance of *Streptococcus* species as major components; however, when it comes to disruption of normal flora, its composition changes according to disease evolution. Normal esophageal microbiota is represented by Gram-positive bacteria from *Bacilliota* phylum and it is known as a Type I microbiome, while Type II encounters Gram-negative bacteria from *Bacteroidota*, *Pseudomonadota* and *Fusobacteria* phylum found in specific pathologies that involve excess acid exposure such as gastroesophageal reflux disease (GERD) and Barrett’s esophagus (BE) [31]. BE is a metaplastic alteration of the normal esophageal mucosa. It appears due to the prolonged acid exposure in GERD. BE is considered to be a predisposing factor for esophageal adenocarcinoma (EAC) [32].

Reflux related disorders are damaging the distal esophageal mucosal barrier with the inflammation of epithelial cells and secondary microbiome dysbiosis, illustrating a shift from the Gram-positive bacteria to Gram-negative and anaerobic colonization, predominantly *Prevotella*, *Haemophilus*, *Neisseria*, *Campylobacter* and *Fusobacterium* genera, some of them not found in the normal esophageal microbiota [31]. It is thought that esophageal microbiota comes from the oral cavity via swallowing or from the stomach, secondary to reflux. One of the pathological mechanisms which can lead to the development of GERD may be the release of biochemical mediators, such as lipopolysaccharides (LPS), a major component of the outer membrane of Gram-negative bacteria that can influence the tonicity of the lower esophageal sphincter. LPS, found in increased amounts in patients with esophagitis and BE, can bind to toll like receptors (TLR4) and other cell surface receptors and induce nitric oxide synthesis with secondary relaxation of the esophageal sphincter, delay the gastric emptying through its production of cyclooxygenase 2 and encourage the expression of inflammatory cytokines [31,32,33]. LPS of Gram-negative bacteria and lipoteichoic acid (LTA) in Gram-positive bacteria are assessed as virulence factors that modulate the host’s innate immune response [34].

Some studies identified a co-exclusion relationship between *Streptococcus* spp., representative for the normal microbiome, and *Prevotella* spp., usually found in acid-exposed environments, while others used the fraction between the two of them as a risk factor for BE evolution [35]. Esophagitis and BE are associated in some cases with the presence of *Escherichia* genus due to its role in promoting inflammation and metaplasia [30,36]. *Campylobacter* species are found only in association with esophageal pathology. IL-18, a proinflammatory cytokine that takes part in tumor progression, records an increased level in patients with *Campylobacter* colonization than in non-colonized ones [37,38]. As for patients with esophageal dysplastic lesions, they have a significant lower number of microbes in the upper GI tract than healthy individuals [39]. EAC is characterized by a decreased number in both Gram-positive and Gram-negative bacteria, but with high levels of *Lactobacillus* spp. when compared to healthy or BE patients [30,31]. Increased lactic acid bacteria could support oncogenesis and its progression by promoting the process of angiogenesis, immune escape, cell migration and metastasis [32]. Nonetheless, the role of the abnormal microbiota in BE’s evolution to EAC is not well understood. It may be a result of chronic inflammation, microbial metabolism or the genotoxicity promoted by the alteration of the microbiota [30,40].

It is generally accepted that gastric acidity, *H. pylori* infection and its eradication treatment have the capacity of influencing the esophageal microbial composition. However, the influence of *H. pylori* infection on BE and EAC is still debated, as different study results are contradictory to each other. It is thought that *H. pylori* gastric colonization would offer protection upon EAC [41,42]. Referring to the bacteria’s eradication, it is known that the esophagus and the stomach microbiota is affected by antibiotic and proton-pump inhibitor (PPIs) treatment, however it is unclear if the PPI effects on the GI flora is beneficial, as their use leads to acid suppression and secondary bacterial overgrowth [30,42]. Probiotics, when given in association to the PPIs treatment, could regulate gut microbiota. A probiotic combination of *Bacteroides subtilis* and *Enterococcus faecium* administrated together with esomeprazole improved abdominal symptoms, decreased bacterial overgrowth in comparison to placebo and lengthened the time to recurrence of the disease. However, there is no proof that PPIs influence dysplasia and the progression of BE to EAC [43].

A lower risk of developing EAC has been observed in individuals with high levels of *Bacilliota*, *Pseudomondota*, *Corynebacterium durum*, *Prevotella nanceiensis*, and *Streptococcus pneumoniae* [44]. Esophageal squamous cell carcinoma (ESCC), on the other hand, has a microbiota represented by an abundance of *Streptococcus* species, *Veillonella parvula*, and *Porphyromonas gingivalis,* while the genera *Lautropia*, *Bulleidia*, *Catonella*, *Corynebacterium*, *Moryella*, *Peptococcus*, *Treponema* and *Cardiobacterium* are found in lower levels than for normal microbiota [39]. As for oral microbiota, pathogens like *Tannerella forsythia* and *Aggregatibacter actinomycetemcomitans* can successfully be used as biomarkers for the identification of EAC. For the therapeutic intervention on flora disruption, studies confirmed that BE progression to EAC could be influenced by the use of probiotics as they might have an inhibitory action on some biomarkers responsible for esophageal carcinogenesis. Probiotics have positive effects when given postoperative in patients with esophageal cancer. Constipation, gastric retention and abdominal meteorism improve after probiotic administration [44].

Eosinophilic esophagitis (EoE) is a chronic immune-mediated disorder affecting the esophagus. Characterized by eosinophil-predominant inflammation, Eoe is mediated by type 2 helper T (Th2) cell activity, primarily triggered by food antigens [45]. The EoE activity seems not to directly affect the bacteria load of the esophageal mucosa but it is associated with changes in microbiota diversity. It involves a shift from a majority of Gram-positive bacteria to an increase in Gram negative bacteria, similar to microbiota changes of composition seen in reflux related disease [31,46]. *Pseudomonadota* such as *Neisseria*, *Corynebacterium* and *Haemophilus* spp. increase their levels in active EoE [31,47,48], independent of treatment status or degree of mucosal eosinophilia [46]. Current studies suggest that the salivary microbiome could be used as a non-invasive marker to monitor pediatric patients with EoE, as endoscopic and histological scores are positively related to the salivary amount of *Haemophilus* spp. [47,49,50]. *Haemophilus* spp. were found in increased levels both in esophageal secretion and mucosal biopsies of untreated EoE patients, but also in salivary samples of children with active EoE, increasing their level with disease activity [50,51]. When analyzing salivary samples of children with active EoE, a tendency to a decrease in microbial richness and alpha diversity was observed. In contrast, specific taxa such as *Streptococcus* spp. seem to increase its amount [50,52]. *Lactococcus lactis NCC 2287*, an inducer of IL-10 immunomodulatory cytokine and an inhibitor of IL-5, a cytokine affecting the survival of eosinophils, has shown its capacity to improve clinical scores in a food allergy model when used as treatment [45]. *Bifidobacterium lactis NCC 2818* was also tested for its capacity to decrease esophageal inflammation in a EoE murine model but its effects were weaker than those obtained after *L. lactis NCC 2287* administration. These results in animal models are proof of the fact that probiotics could be used in decreasing esophageal eosinophilic inflammation [46].

### 3.2. Gastric Pathology

Despite the stomach’s acid environment, a diverse microbiota can be found in vivo. *Pseudomonadota* and *Bacilliota phylum* are predominating the gastric mucus layer, while *Bacteroidota*, *Actinomycetota* and *Bacilliota phylum* are characteristic for the gastric fluid. Usually, bacteria found in the gastric fluid are transient, and are thus non representative for gastric microbiota. As for the normal microbiota, *Veillonella* spp., *Lactobacillus* spp. and *Clostridium* spp. are most frequent in healthy individuals [53,54].

When present, *Helicobacter pylori (H. pylori)* has the highest percentage in the gastric microbiota, both in normal gastric mucosa but in non-atrophic gastritis as well [36]. Several studies have proven that *H. pylori* infection is associated with lower alpha diversity in gastric microbiota, as its oncoproteins can trigger microbial dysbiosis [55]. *H. pylori*-positive individuals’ gastric microbiome is characterized by *Pseudomonadota* (68.7%), *Bacillota* (14.7%), *Bacteroidota* (8.3%), and *Actinomycetota* phylum (6%). The same phylum but in different percentages are identified in *H. pylori*-negative subjects: *Pseudomonadota* (52.6%), *Bacilliota* (26.4%), *Bacteroidota* (12%), and *Actinomycetota* (6.4%) [53,56]. Patients with antral gastritis *H. pylori*-positive present a decrease of *Pseudomonadota* phylum and an increase of *Bacilliota* phylum, while those with atrophic gastritis have an increased *Streptococcus* population and a decrease in *Prevotella* spp. levels. In non-ulcer dyspepsia versus gastric ulcer, the prevalence of non-*H. pylori* bacteria was higher. Following *H. pylori* eradication normal stomach microbiota such as *Bacilliota*, *Bacteroidota*, *Actinomycetota*, *Cyanobacteria*, and *Fusobacteria* increased their levels, similar to healthy individuals’ microbiota, but the baseline status of the microbiota was rehabilitated in the 2 years after the administration of the treatment [49,53,55].

As for the positive effect of bacteria on human microbiota, probiotics products associated with antibiotic treatment have a positive effect on clinical symptomatology, reduce microbiota imbalance and lower the incidence of drugs’ adverse reactions [41]. *Bifidobacterium* and *Lactobacillus* spp. are probiotics with a protective effect related to *Helicobacter* infection. Moreover, their administration shows an improvement in achieving complete *H. pylori* eradication and a reduction in antibiotic therapy’s side effects [54,57]. *Lactobacillus reuteri*, when associated to eradication treatment, seems to inhibit *H. pylori*’s growth and diminish the antibiotic-induced alteration in gastric microbiota. *Lactobacillus acidophilus* and *Lactobacillus bulgaricus* have a similar effect as they decrease bacteria’s adhesion to epithelial cells of the stomach but they also manifest an inhibitory aspect on the IL-8 production of mucosal cells [41,54].

The mixture of Chinese strain *L. acidophilus*, Japanese strain *L. acidophilus*, *S. faecalis* and *B. subtilis* seem to have a good effect on *H. pylori* infection, as it produces substances with an antibacterial role, reduces the growth of the bacteria, prevents bacterial adhesion, inhibits the mucosal inflammation, and helps the gastric mucosa permeability to return to its normal parameters [29].

*Helicobacter pylori*, recognized in 1994 by the World Health Organization (WHO) as a type I carcinogen, is linked to the development of gastric cancer through its virulence factors: cytotoxin-associated gene A (cagA), vacuolating cytotoxin (vacA), and outer membrane proteins (OMPs) [54]. Several studies admit that, since a low percentage of individuals infected with *H. pylori* develop gastric cancer, the presence of the bacteria seems essential but is not enough for the neoplasia to evolve. With regard to carcinogenesis, normal stomach microbiota and *H. pylori* share the same role, with the microbiota changing its composition from non-atrophic gastritis to intestinal metaplasia and gastric cancer (GC) [49,53]. Mucosal atrophy and hypochlorhydria facilitate bacterial overgrowth, and dysbiosis promotes chronic inflammation and regulates many signaling pathways while inflammation leads to tumor progression, invasion and, finally, metastasis [53]. There is a chain relationship between inflammatory cytokines, aberrant DNA methylation, the activation of oncogenes and the inactivation of tumor-suppressor genes. Dysbiosis also triggers a series of innate and adaptive responses related to carcinogenesis, while microbial metabolites share an adjuvant role in the process [42,55]. As for PPIs use, it is known that it encourages microbial growth through its genotoxic potential but, similar to esophageal cancer, it is still uncertain whether it influences the individual risk for gastric cancer or not [44].

Gastric cancer microbiota is described by a lower abundance of *H. pylori* with the increased levels of other bacteria such as *Lactobacillus*, *Enterococcus*, *Carnobacterium*, *Parvimonas*, *Citrobacter*, *Clostridium*, *Achromobacter*, and *Rhodococcus* genera, as well as the oral species *Fusobacterium nucleatum*, *Veillonella* spp., *Leptotrichia* spp., *Haemophilus* and *Campylobacter* spp., similar to non-*Helicobacter* infected patients [42,54]. Lactic acid bacteria (LAB) promote DNA damage secondary to its production of reactive oxygen species (ROS), contributes to mutagenesis, the overexpression of protooncogene, angiogenesis, and inhibits programmed cell death [27]. *Lactobacillus* spp., *Clostridium colicanis* and *F. nucleatum* can be used as biomarkers for GC progression [55].

*Lactobacillus acidophilus*, *Bifidobacterium infantis*, *Bacillus cereus* and *Enterococcus faecalis,* when given in combination to patients with GC, had positive effects on normalizing the gastric microbiota’s physiological composition, reduced inflammation and improved the response of the immune system. *Lactobacillus casei* can have an inhibitory role in GC progression and induce apoptosis, while the fermented milk which resulted from the activity of *Propionibacterium freudenreichii* can induce cell apoptosis but also amplify camptothecin’s cytotoxicity. *Lactobacillus* can also suppress the proliferation of metastatic cells in GC [55,58].

### 3.3. Intestinal Pathology

Irritable bowel syndrome’s (IBS) pathogenesis is not known; however, it has recently been described as a familial predisposition as well as a genetic factor related to the disease. Many shifts of the normal flora have been described, although none is specific to the disease. The majority of patients associate an intestinal bacterial overgrowth, the severity of the symptoms being negatively linked to bacterial diversity [59]. Patients with IBS and diarrhea encountered a reduction of bifidobacteria and lactobacilli and an increase in the *Enterobacter* species, while the ones with constipation reported an increase in *Veillonella* spp. [60]. British Society of Gastroenterology guidelines suggests that probiotics could improve general symptoms as well as abdominal pain, and recommends a 12-week administration, with no reference to a specific strain [61]. IBS patients with diarrhea predominance seem to have achieved a symptom improvement after receiving a probiotic association of *L. acidophilus*, *L. plantarum*, *L. rhamnosus*, *Bifidobacterium breve*, *B. lactis*, *B. longum* and *Streptococcus thermophilus* [62,63].

Intestinal bowel disease (IBD) is associated with gut dysbiosis, however it is not clearly established if the changes in the intestinal microbiome are the true cause or they appear as a result of the disease itself. Individual gene polymorphisms have the ability of influencing the diversity and the composition of the intestinal microbiota, while the abnormal microbiota can activate the immune system with secondary inflammation and disease development, all in the terms of genetic susceptibility [23,24,29,64,65].

IBD individuals display a different GI microbiota compared to healthy individuals, with a reduction in biodiversity associated with modified microbiome metabolite composition and subsequent aberrant immune responses and tissue damage [29,34]. Changes appeared in bacteria with a role in inflammation, either suppressing or promoting it, as part of IBD pathogenesis [17,24]. Several studies performed in IBD patients have encountered lower levels of *Bacilliota* phylum, especially *Faecalibacterium prausnitzii* (*F. prausnitzii*), and an increase in *Bacteroidota* and *Pseudomonadota* phylum such as *Desulfovibrio desulfuricans* (*D. desulfricans*) and *E. coli* [24,34,65]. A decrease of short chain fatty acids (SCFA) producing bacteria (*Clostridium* cluster IV, XIVa, XVII and *F. prausnitzii*) is a particularity of IBD abnormal microbiota. *F. prausnitzii*, a metabolically active commensal bacteria with an important role in the up-regulation of Tregs and anti-inflammatory cytokines, was found in lower amounts in Crohn’s disease (CD) patients compared to healthy individuals [65,66]. Its decreased levels could anticipate CD relapse in patients with remission and an increased risk of recurrence of the disease after surgery [24,67].

In CD, approximately 30% of patients were observed with an abundance of *E. coli* strains (adhesion-invasive *E. coli*-AIEC), causing an amplification of gut permeability and inflammation. It was also found in some ulcerative colitis (UC) patients’ fecal samples [66].

CD microbiota appears to be represented by a reduction in the amount of useful butyrate-producing organisms as *Faecalibacterium* species, *Christensenellaceae*, *Methanobrevibacter* species, and *Oscillospira* species. IBD patients also have larger amounts of sulfate-reducing bacteria. *D. sulfuricans* is one of the hydrogen sulphate producers which can secondarily induce mucosal inflammation. There has also been observed an increase of mucolytic bacteria such as *Ruminococcus* spp. [68,69].

Regarding fungal dysbiosis, patients with IBD displayed compositional differences compared to healthy individuals, although between the CD and UC mycobiome no disturbances were reported [70]. *Candida albicans’* increased levels were associated with remission of the disease, whereas *Candida tropicalis* showed interactions with anti-*Saccharomyces cerevisiae* antibodies, biomarkers associated with CD [70,71].

As for virome alteration, the expansion of the *Caudovirales* family in ileal and gut samples, rather than in the colon samples, was reported in both CD and UC [72]. An inverse relationship was identified between *Caudovirales* and bacterial richness and diversity in IBD [73]. Cornuault et al. reported *F. prausnitzii* phages to be more prevalent or more abundant in IBD patients [74]. Higher levels of *Hepadnaviridae* family transcripts and lower levels of *Polydnaviridae* and *Tymoviridae* were reported in CD, while in UC was an increased abundance of the *Hepeviridae* family and a reduced abundance of the *Virgaviridae* family was identified [75].

As for IBD treatment, the microbiome-immune interface offers some therapeutic opportunities. Probiotics can be used to repopulate flora with anti-inflammatory proprieties, antibiotics and phage therapy can remove overexpressed pro-inflammatory microbiota, while the entire microbiome can be reestablished through fecal transplantation [67]. Probiotics use in CD patients show no effects, but in UC patients seem to have a positive effect on inducing and maintaining remission: the BB12 strain of *Bifidobacterium animalis* given in combination with the LA 5 strain of *Lactobacillus acidophilus* improved the induction and maintained the remission rates of UC, both in single use or as adjuvant therapy [65,76,77]. A combination of four strains of *Lactobacillus* species, three strains of *Bifidobacterium* species and *Streptococcus salivarius* is used to improve immunological tolerance in order to prevent pouchitis in IBD patients. The BB12 strain can prevent UC development by reduction the TNF-α mediated apoptosis of intestinal epithelial cells [66,78].

SCFAs, primarily acetate, propionate, and butyrate are produced from dietary fiber in the gut and have been estimated to provide approximately 60–70% of the energy requirements of colonic epithelial cells [79] *Bacilliota* phylum mainly synthesize butyrate and *Bacteroidota* mainly synthesize acetate and propionate. Butyrate is the major energy source for colonocytes and contributes to the maintenance of intestinal homeostasis. Butyrate activity involves the epigenetic regulation of gene expression through the inhibition of histone deacetylase, has anticarcinogenic and chemo preventive effects, neuroprotective effects, and anti-inflammatory effects, influencing obesity, insulin resistance, cardiovascular diseases, immunoregulation, and inherited disorders [79,80]. Propionate influences lipid synthesis by hepatocytes, is involved in weight control by stimulating satiety and presents anti-carcinogenic effects [81]. Its synthesis is produced by several bacteria from *Bacteroiodota* and *Bacilliota* phylum [82]. Due to their proprieties, SCFAs are successfully used as therapeutic agents. In their study on IBD patients, Facchin et al. reported that sodium butyrate administration increased the amount of SCFA-producing bacteria, enhancing the inflammatory response [83].

The GI motility is mostly under the control of the enteric nervous system, the gut’s own independent nervous system. Gut microbiota has a huge impact on the complex signaling of the enteric nervous system, modulating the motility of the gut [84,85]. Commensal as well as pathogenic bacteria such as *Vibrio cholerae* and *Salmonella typhimurium* can have a particular effect on gut motility [82,86]. Other bacterial species, such as *E. faecalis*, *E. faecium* and *L. brevis,* can produce luminal dopamine, also influencing GI motility [87]. In GI motility disorders, the modulation of the microbiota is thought to have a positive effect. In animal models, the administration of *L. acidophilus* and *B. bifidum* was reported to have a beneficial effect, improving intestinal transit and the contractility of the small intestine [88]. *L. rhamnosus GG* increased choline acetyltransferase expression, responsible for acetylcholine synthesis, the principal metabolite involved in gut motility [89]. *E. coli Nissle 1917* displayed an inhibitory action on the smooth muscle contractility [90]. *B. thetaiotamicron*, given to rodent models, restored the expression of excitatory and inhibitory motor neuron-signaling enzymes [91]. Human studies also reported positive results of probiotic strains on GI motility. *B. longum*, *L. acidophilus*, *S. thermophiles* and *E. faecalis* displayed an inhibitory effect on human colonic muscle [92]. *Akkermansia muciniphila* and *Bacteroides* spp. obtained a modulation of longer gut transit time when given to humans [93]. The increased levels of *Ruminococcus* and *Bacteroides* spp. were linked to shorter transit times. In contrast, the abundance of *Prevotella* was reported to be associated with longer transit times [94]. *L. reuteri* DSM 17938 was successfully used in the management of gastrointestinal symptoms, abdominal pain, diarrhea and constipation, both in adults and children [95]. *B. lactis* HN019TM is another probiotic that can successfully contribute to relieving gut dysmotility-related disorders [96].

Short bowel syndrome (SBS) is a rare disease associated with compromised intestinal function, small intestine bacterial overgrowth (SIBO), mucosal damage and increased permeability [97]. SIBO is commonly characterized by increased levels of aerobic and anaerobic gram-negative bacteria such as *Proteus mirabilis*, *Escherichia coli*, *Klebsiella pneumonia* and *Enterococcus* spp., found frequently in the colon. SIBO might also appear as a shift between normal major phyla proportion [98]. Probiotics could be used in SBS patients to reduce the alteration of the microbiota and to improve the intestinal epithelial and immunological function, but currently there is a lack of evidence supporting systematic use [97,98,99].

Several lactobacilli species are able to produce enzymes used in the digestive process. Among lactobacilli-derived enzymes, lactase, proteases, peptidases, fructanases, amylases, bile salt hydrolases, phytases, and esterases are mentioned. Enzyme-producing lactobacilli strains enhance food digestibility and nutrient bioavailability, decrease malabsorption and intolerance side-effects, and release bioactive molecules with functional properties that cannot be accessed by the human metabolism [96].

Diverticular disease (DD) is a common GI disorder. The alteration of intestinal microbiota and chronic inflammation are potential factors for disease progression [100]. Fecal material stasis leads to diverticular bacterial overgrowth, the impairment of the mucosal barrier function, inflammatory cytokine release, and low-grade inflammation. Microbiota disruption and mucosal inflammation are further associated with dysmotility and abdominal symptoms [100]. Genera including *Enterobacter*, *Streptococcus* and *Bacteroides* seem to be associated with DD, while lactobacilli and bifidobacteria reduce their level when used as a therapeutic option. Patients about to experience an acute episode of diverticulitis display a reduction in taxa with anti-inflammatory activity such as *Clostridium cluster IV*, *Lactobacillus* spp. and *Bifidobacterium* spp. A *Pseudomonadota* phylum increase is also common in the early stages of the disease and might be used as a screening tool for identifying DD at its outbreak [101]. As for DD treatment, the use of rifaximin is associated with abdominal symptom relief but also with changes in microbiota configuration. The genera of *Roseburia*, *Veillonella*, *Streptococcus* and *Haemophilus* decrease their level after rifaximin administration [95].

Lactobacilli use as probiotic strains has reduced bloating and abdominal pain levels in patients with symptomatic uncomplicated DD [102]. *Lacticaseibacillus paracasei* (formerly *Lactibacillus paracasei*) CNCMI1572 (LCDG), a strain normally present in healthy individuals’ microbiota, has been evaluated in several studies. It has an anti-inflammatory effect by modulating many cytokines such as IL-6, IL-8 and IL-10, and has the capacity to modify specific microbial groups, inhibiting bacterial overgrowth seen in DD and increasing SCFA levels with an effect on intestinal homeostasis [100]. *Lactobacillus salivarius*, *Lactobacillus acidophilus* and *Bifidobacterium lactis* are successfully used in the treatment of acute diverticulitis [102]. *L. reuteri* use in DD reduces acute symptoms but also inflammatory marker levels [103].

Normal colonic microbiota suffers some changes in colorectal cancer and it encounters an increased level of *Enterococcus*, *Peptostreptococcus*, *Parvimonas*, *Fusobacterium*, *and Porphyromonas* spp., while *Bifidobacterium*, *Clostridium*, *Lactobacillus*, *Ruminococcus*, and *Roseburia* spp. seem to decrease their numbers [104]. *E. faecalis*, *Fusobacterium nucleatum*, *Escherichia coli*, *Bacteroides fragilis*, *H. pylori* and *S. bovis* are playing an important role in colon carcinogenesis. They can induce chronic inflammation through their metabolites or by invading the normal tissues and generating abnormal cell proliferation [34,44,58].

*E. faecalis* produces chronic inflammation and DNA damage with a role in developing CRC in IBD patients. *F nucleatum*’s identification in colorectal cancer (CRC) patients indicates a high risk of chemoresistance and disease recurrence. It supports inflammation, blocks the immune system and the antitumoral response, and promotes metastatic dissemination [58,69]. Enterotoxigenic *Bacteroides fragilis* (ETBF) is also involved in CRC pathogenesis. When present, it can be used as a predictor of poor outcome. *E. coli*, usually found in colitis-associated cancer, can produce direct DNA damage and genomic instability [58,68]. *H. pylori* infection can lead to CRC as a result of its inflammatory reactions and epithelial damage. Through its gastrin secretion it can promote the increased proliferation of colon mucosal cells and elevate the risk of CRC [47,104]. *S. bovis* antigen induces tumor cell proliferation and angiogenesis and has an inhibitory effect on apoptosis [42,104].

The CRC enriched bacteria including *Bacteroides fragilis*, *Escherichia coli*, *Enterococcus faecalis*, *Streptococcus gallolyticus*, *Peptostreptococcus anaerobius*, *Fusobacterium nucleatum*, *Parvimonas micra*, *Porphyromonas asaccharolytica*, and *Prevotella intermedia* act as promising biomarkers for the early detection of intestinal cancer [105].

*Parvimonas micra*, a commensal of the oral cavity, is strongly associated with CRC, as it has been found in increased amounts in CRC patients’ tumoral tissue and feces. Its presence is also correlated to a poor survival rate [106]*. P. micra* can activate the central transcription factor NF-κB, a key modulator of inflammation and immune responses with an important role in CRC carcinogenesis. Recently, it has been shown to be associated with the CMS1 subtype of CRC, a subtype characterized by a strong immune cell infiltration of CD8+ cytotoxic T cells, CD4+ T helper and natural killer cells [107]. Zhao et al.’s analysis showed that *P. micra* promotes CRC growth by enhancing Th-17 mediated immune responses [108].

Xia et al. observed an association between the enrichment of *F. nucleatum* or *Parvimonas* spp. and promoter methylation of several tumor suppressor genes (TSGs) in tumor tissues [109]. Studies found *P. micra* to be correlated to *F. nucleatum* in feces, as they might interact to create a pro-inflammatory environment [106,107,108]. In vitro, the two of them have been shown to have the ability to aggregate and form biofilms [106].

LPS produced by *Klebsiella pneumoniae* and other enterobacteria have been shown to have an inhibitory effect on the host tumor suppressor p53 pathway [110]. *E. coli*, usually found in colitis-associated cancer, can produce direct DNA damage and genomic instability [58,68]. *Salmonella* spp. promotes colonic tumorigenesis dependent on its protein, AvrA, which can activate both the Wnt/β-catenin and STAT3 signaling pathways in colonic tumor cells [110].

In CRC, bacteriophages including *Siphoviridae* and *Myoviridae* allowed the colonization of *F. nucleatum*, bacteria with an important role in CRC tumorigenesis [111]. Nakatsu et al. reported an enrichment of *Inovirus* and *Tunalikevirus* in CRC development, with four taxonomic markers associated with the reduced survival rate [112]. *Trichosporon* spp. and *Malassezia* spp. were found in increased levels in CRC patients’ fecal samples [113]. Similar, Coker et al. reported an increased proportion of *Malasseziomycetes* and decreased levels of *Saccharomycete*s [114].

Studies have shown that probiotic supplements can play a role in the prevention of CRC, as lactic acid producing bacteria can avoid DNA damage and carcinogenesis in high-risk patients. *Bifidobacterium* spp. administration seems to improve the response to immunotherapy or chemotherapy, while a mixture of six strains of *Lactobacillus* spp. and *Bifidobacterium* spp. given for a six-month period to CRC patients after colorectal resection reduced their pro-inflammatory protein levels [104,115,116]. A similar study has shown the improvement in the immune-responsive activities of T cells, macrophages and natural killer cells. Associated with chemotherapy and radiotherapy, *Lacticasei bacilli* reduced the amount of side effects [117].

Celiac disease (CeD) is an autoimmune disorder triggered by gluten and related prolamins that occurs in genetically predisposed individuals [118]. Genetic susceptibility and gluten ingestion do not have the capacity of inducing the disease alone, suggesting that several additional factors might interfere with disease pathogenesis [119,120,121]. Intestinal dysbiosis in CeD has been the subject of different studies as the major environmental factor involved in CeD underlying mechanisms. “Beneficial bacteria” such as bifidobacteria, clostridia and lactobacilli encountered lower levels than in healthy individuals, while “potential pathogenic bacteria” such as *E. coli* and bacteria from *Bacteroidota* phylum increased their amounts [119]. Dysbiosis can also appear as a result of the gluten-free diet (GFD) that eliminates dietary carbohydrates resources used by beneficial bacteria as energy sources [119,120].

Fungal dysbiosis has also been described in patients with CeD [122]. Alpha diversity for fungal species between CeD patients’ sample and healthy individuals had a similar diversity, a beta diversity identifying an overlap of samples from both categories. The *Saccharomycetaceae* family and Saccharomyces cerevisiae were more abundant in CeD patients while *Ascomycota* phylum had notably decreased levels [119]. Similar, high anti-*Saccharomyces boulardi* antibodies were reported in CeD patients with a complete reduction or an important reduction during a gluten free diet (GFD) [119]. *Candida albicans* hyphal wall protein 1, containing identical or highly similar amino acid sequences related to alpha-gliadin and gamma-gliadin T-cell epitopes, might suggest that candida could play a role in CeD initiation and evolvement [119,123]. As for the virome component, current literature suggests that in susceptible individuals, RNA and DNA eukaryotic viruses’ infection may generate a transient disease with secondary loss of tolerance to gluten and CeD development [120]. Meanwhile, bacteriophages could cause CeD either directly or by modulating the normal GI microbiota to a CeD-specific composition [120,122].

In addition to a GFD, patients with CeD can benefit from probiotic supplements. *Lactobacillus rhamnosus* can improve the function of the intestinal barrier, *Bifidobacterium* strains have anti-inflammatory proprieties, while *Lactobacillus* strains assure gluten degradation and reduce gluten concentration and toxicity [118,124]. The combined probiotics of two *Bifidobacterium* spp. strains associated with GFD seem obtain a microbiota similar to healthy individuals’ fecal microbiota [124].

The main changes in human microbiota composition, related to different digestive disorders, are summarized in Table 1.

## 4. Limitations of Microbiome Studies

Although microbiological sciences benefit from numerous advances in modern technology and an extensive characterization of the microbiome has been performed in recent years, there are still limitation that need to be overcome. Variation in study design as well as confounding variables in different studies frequently result in discordant results. The available experimental and bioinformatics methods leave space for bias and unreliable results [125]. There is also a lack of compatibility between existing databases, mainly because there is not a correct scale to be used when comparing the taxonomy and the functions associated to a microbiome [126]. There is also the aspect of the absence of a complete and comprehensive set of microbiome samples and metadata about them to be available for public use. The need for consistency and uniform results will probably lead to the development of new artificial intelligence tools and to their implementation in current medical practice in a global network with a universal database.

## 5. Conclusions

The GI microbiome is highly complex and still considered as a pinnacle of a closely associated relationship with humans’ health status. In this literature review, we have strongly presented the facts and the changes in composition and functional capacity of GI microbiota, which could lead to several GI related diseases. Despite all the advancements in medical technology and molecular techniques, we should further explore the intricacies of the human as a host and its microbiota. Although we clearly understood that GI microbiota influences hosts’ physiology, metabolism and immune status, to prove a dysbiosis as a single causative factor of digestive related pathologies we need extensive research and studies. A better understanding of the relationships between microbiota disruption and disease evolution will lead to the development of new appropriate therapeutic options based on the modulation of the GI microbiota.

## Figures and Tables

**Table 1:** Microbiota changes in digestive disorders.

Disease	Microbiota Changes	References
Barret esophagus	*↑Fusobacterium* spp. *↑Prevotella/Veillonella* spp. *↑Peptostreptococcus* spp. *↑Atopobium* spp. *↓Neisseria/Streptococcus* spp. *↓Aggregatibacter/Campylobacter* spp. *↓Treponema/Corynebacterium* spp. *↓Lactovibrio* spp. ↓Alpha diversity	[30,31,33,36]
Esophageal adenocarcinoma	*↑Lactobacillus fermentum* *↑Prevotella* spp. *↑Enterobacter* spp. *↑Akkermansia muciniphila* *↓Streptococcus pneumoniae* *↓Alpha diversity*	[30,31,40,41,44]
Esophageal squamous cell carcinoma	*↑Streptococcus* spp. *↑Veillonela parvula* *↑Porphyromonas gingivalis* *↑Fusobacterium* spp. *↓Lautropia/Bulleidia* spp. *↓Catonella/Corynebacterium* spp. *↓Moryella/Peptococccus* spp. *↓Treponema/Cardiobacterium* spp. ↓Alpha diversity	[39]
Eosinophilic esophagitis	↑*Neisseria/Corynebacterium* spp. *↑Veillonella/Prevotella* spp. ↑*Haemophilus* spp. ↓*Parvimonas/Porphyromonas* spp. ↓*Streptococcus* spp. ↓Alpha diversity	[31,46,47,48,49,50,51,52]
H. pylori infection	↑*Lactobacillus* spp. ↑*Actinomyces/Gemella* spp. ↑*Streptococcus* spp. ↑*Haemophilus* spp. ↓*Clostriudium* spp. ↓Alpha diversity	[53,54,55,56,57]
Gastric cancer	↑*Lactobacillus/Enterococcus* spp. ↑*Parvimonas/Citrobacter* spp. ↑*Clostridium/Achromobacter/Rhodococcus* spp. ↑*Bacteroides fragilis/Akkermansia muciniphila/Fusobacterium nucleatum* *↑Veillonella/Leptotrichia/Haemophilus* spp. ↑*Campylobacter/Streptococcus* spp. ↓*Helicobacter* spp. ↓*Neisseria* spp. ↓*Acinetobacter* spp. ↓Alpha diversity	[42,54,55]
Inflammatory bowel disease	↑*Desulfovibrio desulfuricans* ↑*Escherichia coli* ↑*Ruminococcus* spp. ↑*Fusobacterium* spp. ↓ *Faecalibacterium prausnitzii* ↓ *Clostridium cluster IV* ↓*Clostridium cluster XIV*	[24,34,65,67,68,69]
Short bowel syndrome	↑*Lactobacillus* spp./*Prevotella* spp. ↓C*lostridium*/*Bacteroides* spp. ↓Alpha diversity	[97,98]
Diverticular disease	↑*Akkermansia* spp. ↑*Enterobacter* spp. ↑*Streptococcus* spp. ↓*Lactobacillus* spp. ↓*Clostridium cluster IV*	[95,100,101]
Colorectal cancer	↑*Fusobacterium nucleatum* ↑*Bacteroides fragilis* ↑*Escherichia coli* ↑*Campylobacter/Porphyromonas* spp. ↑*Streptococcus* spp. ↑*Leptotrichia/Sutterella* spp. ↓*Clostridium cluster IV* ↓*Clostridium cluster XIV* ↓Alpha diversity	[34,44,58,68,69,104,105]
Celiac disease	↑*Prevotella* spp. ↑*Clostridium* spp. *↑Atopobium* spp. ↓*Lactobacillus/Bifidobacterium* spp.	[118,119,120,121]
